# Expanding the clinical utility of liquid biopsy by using liquid transcriptome and artificial intelligence

**DOI:** 10.1016/j.jlb.2024.100270

**Published:** 2024-10-16

**Authors:** Maher Albitar, Ahmad Charifa, Sally Agersborg, Andrew Pecora, Andrew Ip, Andre Goy

**Affiliations:** a Genomic Testing Cooperative, Lake Forest, CA, USA; b John Theurer Cancer Center, Hackensack, NJ, USA

**Keywords:** Liquid biopsy, cell-free DNA (cfDNA), cell-free RNA (cfRNA), Cancer diagnosis, Next generation sequencing, Artificial intelligence (AI)

## Abstract

Most of the current utilization of liquid biopsy (LBx) is based on analyzing cell-free DNA(cfDNA). There is limited data on using cell-free RNA (cfRNA) levels (liquid transcriptome) in LBx. The major hurdles for using liquid transcriptome is its low level in circulation and the dilutional effects of various tissues that may pour their RNA into circulation. We explored the potential of using artificial intelligence (AI) to normalize the cancer-specific cfRNA and to enable liquid transcriptome to predict diagnosis. cfRNA transcriptomic data from 1009 peripheral blood samples was generated by hybrid capture next generation sequencing (NGS). Using two-thirds of samples for training and one third for testing, we demonstrate that AI is able to distinguish between normal control (N = 368) and patients with solid tumors (N = 404) with AUC = 0.820 (95 % CI: 0.760–0.879), patients with myeloid neoplasms (N = 99) with AUC = 0.858 (95 % CI: 0.793–0.924) and patients with lymphoid neoplasms (N = 128) with AUC = 0.788 (95 % CI: 0.687–0.888). Specific diagnosis was also possible when patients with lung, breast, colorectal, and myelodysplastic subgroups were tested. This data suggests that liquid transcriptomics when used with AI has the potential of transforming “liquid biopsy” to “true” biopsy, replacing the need for tissue biopsy.

## 1 Introduction

Liquid biopsy (LBx) has become an essential test for the diagnosis and monitoring of patients with various types of cancer [[1], [2], [3], [4]]. Clinical applications of LBx are growing rapidly. LBx was initially established for hard-to-biopsy patients due to the location of the cancer or due to the presence of significant co-morbidity. Currently LBx is used for early detection or confirmation of the presence of cancer, detecting the presence of actionable alterations, predicting response or resistance to therapy as well as prognosis and survival after immunotherapy or targeted therapy [[5], [6], [7], [8]]. Most of current LBx testing is based on analyzing circulating cell-free DNA (cfDNA). This includes mainly next generation sequencing (NGS), methylation analysis, and fragmentomic analysis [[9], [10], [11]]. However, the utilizing of circulating cell-free RNA (cfRNA) is emerging as a reliable approach [12]. LBx that combines the analysis of cfDNA with cfRNA provides more comprehensive evaluation of molecular abnormalities [12]. Higher sensitivity in detected mutations has been reported when cfRNA is used [12]. However, when the mutations lead to early termination of protein synthesis, which is typically associated with RNA instability, RNA is less sensitive than DNA in detecting mutations [12]. cfRNA also improves detection of fusion genes and chromosomal translocation [13]. More importantly, cfRNA sequencing by NGS provides quantitative expression data of genes and opens the door for utilizing this transcriptomic data to better understand the cancer itself as well as the host [[14], [15], [16]]. cfRNA transcriptomic data has the potential to provide information on the immune system, the level of cytokines and chemokines that are generated by the tumor as well as by the host.

Except for rare cases where tumor burden is very high, the level of cfRNA is very low. Furthermore, it is expected that cfRNA generated by cells other than tumor cells will also be present in peripheral blood. Normal hematologic cells, endothelial cells, and reactive cells responding to the tumor might pour their RNA into circulation to be mixed with RNA generated by tumors. In principle, tumor cells have higher turnover and the cfRNA is relatively more enriched by tumor-specific cfRNA, but this may vary with the stage of the cancer as well as the type of cancer [17]. To focus on the tumor-specific cfRNA, normalization is required and relative levels rather than absolute levels should be the focus of the analysis. Such normalization should consider multiple biomarkers that correct for various conditions that are physiologic as well as the results of the neoplastic process. For this reason, the use of artificial intelligence (AI) is essential for this normalization [18]. Supervised training of AI using large numbers of cases that encounter various physiological and reactive processes is essential for proper development of algorithms that can take advantage of liquid transcriptomics.

We describe in this paper the importance of liquid transcriptomics in LBx, especially in the diagnosis of various types of tumors. We also show that using AI is necessary for the interpretation and utilization of liquid transcriptomics in this diagnosis and classification.

## 2 Results

### 2.1 Stand-alone cfRNA data in cancer diagnosis

In some cases, simple evaluation of the cfRNA levels can be adequate for diagnosis. Table 1 shows an example of such cases. Only biomarkers relevant for diagnosis are listed in Table 1. Comparing the tested patient with a normal control, the cfRNA transcriptomic data clearly support the diagnosis of multiple myeloma in a fashion similar to that expected from flow cytometry evaluation. As in flow cytometry, there is significant increase in CD138, CD38 and BCMA expression without significant increase in B-cell markers (CD19, CD20 and CD22).

**Table 1:** Example of levels of various biomarkers detected in cfRNA from normal control and a patient with multiple myeloma.

Biomarkers	Normal control (TPM)	Patient (TPM)		Biomarkers	Normal control (TPM)	Patient (TPM)
BCL2	77	267		CD8B	9	48
BCL6	247	83		FCGR1A(CD64)	63	26
CCND1	257	2043		FGFR3	10	15
CD14	83	63		IRF4(MUM1)	54	270
CD19	28	30		KIT	5	14
CD2	99	47		MAF[t(14; 16)]	68	28
CD22	178	48		MAFB[t(14; 20)]	184	40
CD274 (PD-L1)	6	15		MKI67	125	139
CD33	91	54		MME(CD10)	17	4
CD34	74	22		MS4A1(CD20)	206	33
CD38	54	285		MYC	140	79
CD3D	40	41		NCAM1(CD56)	12	58
CD3E	60	17		NSD2(WHSC1), t(4; 14)	130	97
CD3G	6	8		PTPRC(CD45)	423	99
CD4	55	27		SDC1(CD138)	22	221
CD8A	11	18		TNFRSF17(BCMA)	27	328

**Fig. 1:**
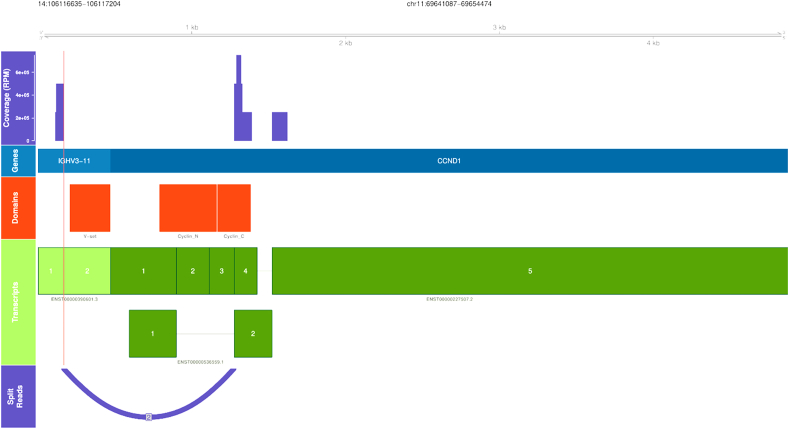
IgH::CCND1 fusion detected in cfRNA of a patient with multiple myeloma.

Furthermore, there is a significant increase in CCND1 mRNA suggesting promoter hijacking. IgH::CCND1 fusion cfRNA was confirmed when fusion analysis was performed (Fig. 1). The data also rules out abnormalities involving MAF, MAFB, MYC or NSD2/FGFR3, which are chromosomal translocations clinically relevant for multiple myeloma diagnosis. Furthermore, B-cell clonality was confirmed in this sample by demonstrating dominance of expression of IgHV 3–21 and IgKV 2–28. The expression of these families was almost 20 folds higher as compared with next expressed immunoglobin family (data not shown).

Adding mutation profile to the expression data can also help in the diagnosis in most cases. However, in numerous cases, the changes in cfRNA are more subtle and not obvious. In these cases, AI is required. Based on our data and our referral pattern, approximately 10 % of cases can be diagnosed based on stand-alone cfRNA and 90 % of cases require AI help.

### 2.2 cfRNA and AI-aided multi-cancer diagnosis

When the level of changes in cfRNA are low as compared to normal control, help from AI is essential for proper diagnosis. This is particularly relevant in order to adjust for various physiological reactive processes. Changes in cfRNA due to infection, inflammatory processes not relevant to cancer, drug therapy and other causes may influence cfRNA levels and AI must adjust for these circumstances. Therefore, the larger the number of samples in training the AI, the better the reliability of the system. Furthermore, the AI system must deal with the high dimensionality that is typically seen in transcriptomic data.

To address the high dimensionality, we implemented a two-step AI approach for arriving at diagnosis using cfRNA. In the first step, we used Bayesian algorithm for ranking and selecting the genes that are relevant for the diagnostic classification. In the second step, we used random forest to select the number of biomarkers needed from the highly ranked genes and to use them in predicting diagnosis.

**Fig. 2:**
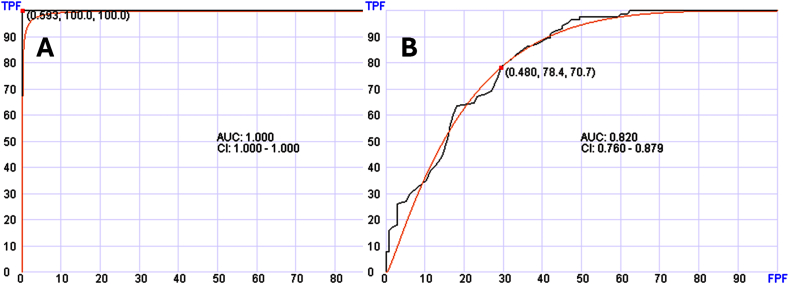
Receiver operating characteristic (ROC) curves for distinguishing solid tumor patients from normal control. Panel A shows the ROC and AUC in the training set. Panel B shows the testing set. TPF, true positive fraction (sensitivity); FPF, false positive fraction (specificity).

We first used this approach to distinguish between normal individuals and patients with various types of solid tumors. Using 269 solid tumor samples and 179 control samples in training, AUC distinguished between the two groups at 100 %. In a testing set of 134 solid tumor samples and 89 normal controls, the AUC was at 0.820 (95 % confidence interval (CI): 0.760–0.879). (Fig. 2). Random forest used 250 genes in this algorithm.

**Fig. 3:**
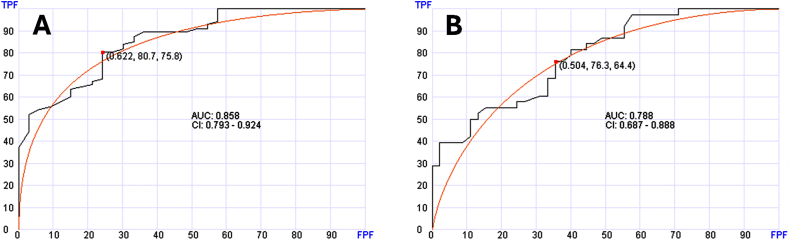
Receiver operating characteristic (ROC) curves of the testing sets for distinguishing patients with hematologic neoplasms from normal control. Panel A shows the ROC and AUC for distinguishing myeloid neoplasms. Panel B shows the ROC and AUC for lymphoid neoplasms. TPF, true positive fraction (sensitivity); FPF, false positive fraction (specificity).

Distinguishing between patients with myeloid neoplasms (99 samples) and normal control was possible using the same approach. The training set using two-thirds of samples showed AUC of 100 % while the testing set showed AUC of 0.858 (95 % CI: 0.793–0.924) (Fig. 3A). Similarly, distinguishing lymphoid neoplasms (138 samples) from normal control using the same AI approach was possible with AUC of 0.788 (95 % CI: 0.687–0.888) in the testing set (Fig. 3B).

### 2.3 cfRNA and AI-aided specific cancer diagnosis

**Fig. 4:**
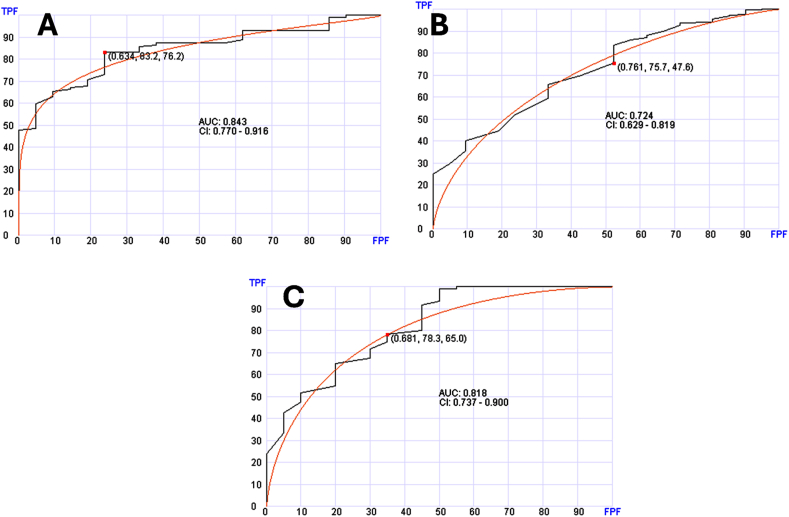
Receiver operating characteristic (ROC) curves of the testing sets to distinguish patients with specific solid tumor. Panel A shows distinguishing breast cancer from other solid tumors. Panel B shows distinguishing breast cancer from entire samples (normal, solid tumors and hematologic neoplasms). Panel C shows distinguishing colorectal cancer from the rest of solid tumors. TPF, true positive fraction (sensitivity); FPF, false positive fraction (specificity).

We also explored if cfRNA can be used in determining the specific cancer diagnosis rather than the broad diagnosis presented above. Of the 404 solid tumor samples, 68 cases were breast cancer. Using the same AI approach, we tested if these breast cancer samples can be diagnosed and separated from the rest of the solid tumor cases. Fig. 4A shows that breast cancer patients can be diagnosed with AUA of 0.843 (95 % CC: 0.770–0.916) in the testing set. The algorithm needed only 14 genes (WDFY3, ZNF585B, PALB2, BMPR1A, POLR2H, PDCD11, THADA, ACSL3, LMO7, WDR70, GOLGA5, FZD6, MSH3, SH3D19) in this algorithm. Distinguishing the breast cancer patients from the entirety of samples (normal, hematologic and solid tumors) (N = 1009), was possible but less reliable (AUC = 0.724, 95 % CI: 0.629–0.819) using 30 genes (Fig. 4B).

Distinguishing the colorectal tumors (N = 61) from the rest of the solid tumors was also possible using 80 genes with AUA of 0.818 (95 % CI: 0.737–0.900) (Fig. 4C). In contrast, distinguishing these colorectal tumor cases from the entire set of tested samples, was not reliable (AUA = 0.660).

**Fig. 5:**
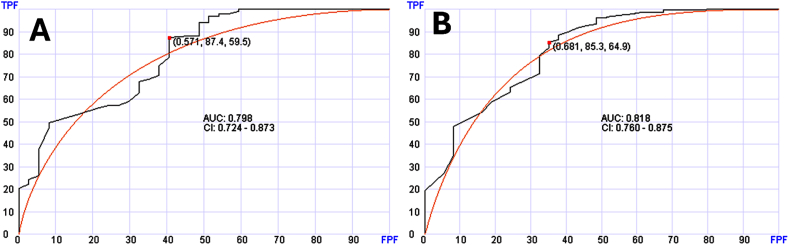
Receiver operating characteristic (ROC) curves of the testing sets to distinguish patients with lung cancer. Panel A shows distinguishing lung cancer from other solid tumors. Panel B shows distinguishing lung cancer from entire samples (normal, solid tumors and hematologic neoplasms).

There were 114 cases of lung cancer among the solid tumor cases. Using the same AI approach and 60 genes, lung cancer was distinguished with AUC of 0.798 (95 % CI: 0.724–0.873) (Fig. 5A). Distinguishing lung cancer diagnosis from the entire group was also possible using 80 genes with AUC of 0.818 (95 % CI: 0.760–0.875) (Fig. 5B).

**Fig. 6:**
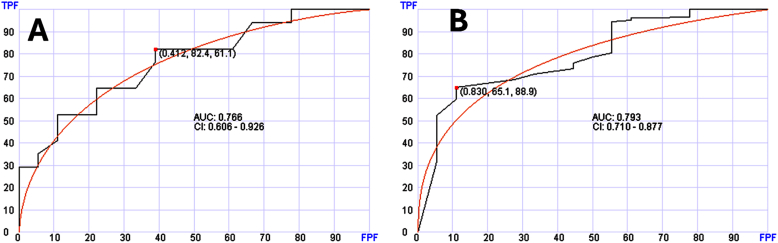
Receiver operating characteristic (ROC) curves of the testing sets to distinguish patients with (myelodysplastic syndrome (MDS). Panel A shows distinguishing MDS from other myeloid neoplasms. Panel B shows distinguishing MDS from entire samples (normal, solid tumors and hematologic neoplasms).

For hematologic neoplasms, a specific cancer diagnosis was also possible. There were 56 samples with MDS among the myeloid cases. Distinguishing MDS from the rest of the myeloid was possible using 40 genes with AUC of 0.766 (95 % CI: 0.606–0.926) (Fig. 6A). MDS cases were also distinguishable from the entire set of samples using 30 genes with AUC of 0.793 (95 % CI; 0.710–0.877) (Fig. 6B).

## 3 Discussion

Neoplastic processes are typically characterized by increased cell growth and high rate of cell turn-over pouring into circulation the content of cellular elements in various forms including microsomes, microvessels, apoptotic bodies and debris [19,20]. These products contain cfDNA and cfRNA which are also called circulating tumor DNA (ctDNA) and circulating tumor RNA (ctRNA). Since cfRNA is more labile and easily degraded, most of the investigational work in this field has been focusing on cfDNA. Recent data shows that cfRNA which might be degraded still can be studied and quantified reliably using NGS. NGS is typically based on sequencing small fragments of nucleic acid and not compromised when cfRNA is fragmented [19]. In this paper, we demonstrate that the levels of cfRNA as determined using NGS can be used in the clinical diagnosis of solid tumors and hematologic neoplasms.

We used a targeted RNA panel and evaluated the expression levels of 1,600 genes. Using a targeted panel of genes increases the dynamic range of the levels of cfRNA and avoids highly expressed genes that may overshadow the genes that are important in the neoplastic process. The selected genes are not only relevant for oncogenesis but also for diagnosis covering most of the genes typically used in flow cytometry analysis, immunohistochemistry and therapeutic approaches. This approach allowed us to establish diagnosis in some cases by simply inspecting the levels of some genes in a fashion similar to that used by flow cytometry or immunohistochemistry testing. We present an example of multiple myeloma and show that diagnosis can be easily established by a pathologist familiar with the characteristics of plasma cells when flow cytometry is used. Furthermore, cfRNA provided information on chromosomal translocation (fusion genes) replacing the need for FISH (Fluorescent in situ hybridization) studies and information on clonality replacing the need for specialized molecular study to establish B-cell clonality.

However, more frequently, the level of cfRNA is not easily delineated from normal control and tumor biomarkers cannot be reliably evaluated. In these cases, diagnosis must be based on overall pattern of expression. More importantly this pattern must account for all possible physiologic or reactive conditions that a patient might be exposed to at the time of collecting the blood sample. This includes infection, nutritional conditions, age-related conditions, and others. The multidimensional nature of transcriptomic data is ideal for this approach assuming appropriate machine learning algorithms and adequate representative samples for training are available. Based on these principles, we first evaluated normal vs patients with broad diagnosis. We explored the diagnostic potential of cfRNA in distinguishing patients with any solid tumor, patients with various myeloid neoplasms, or patients with lymphoid neoplasms vs normal control. As shown in Fig. 2, Fig. 3, AI reliably distinguished patients with these abnormalities from normal control. We used Bayesian approach for feature selection and ranked the analyzed genes based on their ability to distinguish between the tested two diagnostic classes, then we used random forest to establish an algorithm using the Bayesian-selected top-ranked biomarkers for predicting diagnosis.

While distinguishing between the major diagnostic classes is important, we explored the potential of this approach in predicting the diagnosis of specific cancer type. We selected neoplasms presented with a good number of cases in our data set. In our solid tumor data set, breast, colorectal and lung cancers had good number of cases that can be used for training and testing. We explored if reliability of diagnosis can be enhanced when the diagnosis is first established based on the broad diagnosis as described above versus when all samples are considered without triaging. As shown in Fig. 4, Fig. 5, specific diagnosis can be reliably established after triaging into the subgroups. Without triaging diagnosis was also possible and reliable for breast cancer and lung cancer but not colorectal cancer. This suggests that two-step diagnosis is an approach that has the potential of refining diagnosis in some cases.

In hematologic neoplasms, the number of cases was limited but we were able to test cases with MDS diagnosis and demonstrate that specific diagnosis can be established with and without triaging (Fig. 6).

The data clearly shows the value of liquid transcriptomics when combined with AI in the diagnosis and evaluation of patients with cancer. This non-invasive biopsy is clinically very valuable. Combining cfDNA with cfRNA data can transform liquid biopsy from being a molecular tool to find actionable mutations for targeted therapy or for determining minimal residual disease (MRD) to a tool that thoroughly evaluates the neoplastic process in patients with cancer. Adding cfDNA findings to transcriptomic data can strengthen the diagnostic and clinical value of LBx. cfRNA transcriptomic data when added to cfDNA data makes liquid biopsy an actual biopsy that can provide information similar to those expected from tissue biopsy including information typically obtained by flow cytometry and IHC. In this paper we addressed diagnosis and classification, but the same approach has the potential to be used in predicting response, side effects or survival.

The current study is strictly targeting cancer but liquid transcriptomics when combined with AI has the potential to be used to predict non-cancer diseases, especially immune-related or inflammatory and degenerative diseases. However, performing transcriptomic analysis of cfRNA in circulation is not standardized at this time and more studies are needed to understand the technical and clinical factors that may affect the accuracy and reliability of quantifying cfRNA. As discussed above, performing whole transcriptome vs targeted transcriptome may make a difference in the quantification of the RNA due to the fact that the highly expressed genes in the whole trascriptome data may overshadow the genes that are expressed at low levels. The type of tubes used in collecting blood samples and the time interval in processing these tubes may also play a role in preserving cfRNA and may affect reliability of testing. Furthermore, the type of chemistry used in sequencing and the analytical tool used in RNA quantification can also play a role in these studies.

In conclusion, liquid transcriptomics adds a whole new capability to liquid biopsy. In some patients, it may provide data replacing the need for flow cytometry or IHC, but when implemented with AI, it allows us to provide reliable information on specific diagnosis and has the potential of providing information on response and side effects. Testing that incorporates liquid transcriptomics with AI and cfDNA data fulfills the promise of the term “liquid biopsy” and makes it an actual biopsy. However, further studies and confirmation using prospective clinical trials is needed.

## 4 Methods

### 4.1 Patients and samples

Consecutive 1009 peripheral blood samples were used for the study. This included 368 normal control, 404 samples from patients with various types of solid tumors, 138 samples with lymphoid neoplasms and 99 samples from patients with myeloid neoplasms. The 368 normal control included 153 patients with low level mutations consistent with CHIP (clonal hematopoiesis of indeterminate potential). The solid tumors included 26 ovarian, 42 prostate, 27 pancreas, 68 breast, 61 colorectal, and 13 endometrial. The myeloid included 56 patients with myelodysplastic syndrome. The rest were mixed low-level chronic and acute myeloid neoplasms. The lymphoid neoplasms included 12 chronic lymphocytic leukemia and 12 diffuse large B-cell lymphoma. The rest were mixed low-level lymphoid neoplasms including mantle, marginal, T-cell lymphoma and others. The samples were collected during routine liquid biopsy clinical testing. All samples were collected in EDTA (ethylenediaminetetraacetic acid) tubes and processed within 72 h of collection. Diagnosis was established based on clinical history, mutation profile and comparison with tissue-based diagnosis. The study protocol was approved by the Western Copernicus Group Institutional Review Board (New England IRB, Aspire IRB, and Midlands IRB) (Number 1-1476184-1). Patient informed consent was waived due to incidental collection and lack of risk. This study was conducted in accordance with the principles of the Declaration of Helsinki and its later amendments.

cfRNA was extracted from peripheral blood samples collected in EDTA tubes within 72 h of collection. Extraction was performed as previously described [12]. RNA was sequenced using hybrid capture approach of a targeted 1,600 gene panel that includes all immunoglobulin heavy and light chain genes and all T-cell receptors genes. Sequencing was performed using illumina NovaSeq 6000 instrument. More than 80 million reads was required for accepting results. The required percentage of spliced reads was above 20 %. Paired-end 100 base sequencing was performed. The expression levels are calculated based on transcript per million (TPM). In addition to expression levels, the cfRNA sequencing data was also analyzed for mutation profile using the Dragen v3.10.8 – Somatic pipeline. Data also analyzed for fusions and clonality.

## 5 Artificial intelligence model

The cfRNA expression levels of the sequenced genes were used to distinguish between diagnostic classes using AI approach based on first ranking the sequenced genes using Bayesian statistics as previously described then using random forest selecting the top-ranked genes to deliver the best algorithm for predicting the diagnostic class. Two thirds of samples were used for the training of the random forest and one third is used for the testing. Training and testing samples were selected randomly.

## Data availability statement

The data presented in this study are available on request from the corresponding author.

## Ethical approval

The study protocol was approved by the Western Copernicus Group Institutional Review Board (New England IRB, Aspire IRB, and Midlands IRB) (Number 1-1476184-1). Patient informed consent was waived due to incidental collection and lack of risk. This study was conducted in accordance with the principles of the Declaration of Helsinki and its later amendments.

## Funding statement

Funding was provided by Genomic Testing Cooperative.

## Declaration of competing interest

The authors declare the following financial interests/personal relationships which may be considered as potential competing interests:

Maher Albitar reports financial support and article publishing charges were provided by genomic Testing Cooperative. Maher Albitar reports a relationship with Genomic Testing Cooperative that includes: board membership. Maher Albitar reports a relationship with Genomic Testing Cooperative that includes: employment and equity or stocks. Maher Albitar has patent pending to Genomic Testing Cooperative. No If there are other authors, they declare that they have no known competing financial interests or personal relationships that could have appeared to influence the work reported in this paper.
